# Pharmacological Potentiality of Bioactive Flavonoid against Ketamine Induced Cell Death of PC 12 Cell Lines: An In Vitro Study

**DOI:** 10.3390/antiox10060934

**Published:** 2021-06-09

**Authors:** Chintha Venkataramaiah, Bandila Lakshmi Priya, Sholapuri Payani, Jangampalli Adi Pradeepkiran

**Affiliations:** 1 Division of Molecular Biology, Department of Zoology, Sri Venkateswara University, Tirupati 517 502, Andhra Pradesh, India; s.payani63@gmail.com; 2 Department of Zoology, Faculty of Humanities and Sciences, Sri Venkateswara Vedic University, Tirupati 517 502, Andhra Pradesh, India; 3 Department of Biotechnology, Sri Venkateswara Institute of Medical Sciences, Tirupati 517 502, Andhra Pradesh, India; priyaramana386@gmail.com; 4 Department of Internal Medicine, Texas Tech University of Health Science Centre, Lubbock, TX 79430, USA

**Keywords:** PC 12 cell lines, cell viability, LDH assay, ROS assay, clonogenic assay, RT-PCR, neuroprotection

## Abstract

During the past few years, there has been exponential growth in the field of ethnopharmacology in the treatment of different human ailments, including neurological disorders. In our previous study, we isolated, characterized, and reported a novel bioactive compound with therapeutic efficacy in vivo, which was used in the current study. This study was designed to investigate the pharmacological effect and therapeutic mechanism of the natural plant compound 3-(3,4-dimethoxy phenyl)-1-(4-methoxy phenyl)prop-2-en-1-one against ketamine-induced toxicity in PC 12 cell lines. Cell death was induced in PC 12 cell lines by incubating with ketamine, and the protection offered by the compound at different concentrations was studied during pretreatment. The therapeutic efficacy was screened through MTT assay, LDH assay, DCF-DA assay, clonogenic assay, RT-PCR, and densitometric analysis. The bioactive compound caused a significant elevation in cell viability up to approximately 80%, down-regulation of cell damage, reduction in free radical damage caused by intracellular reactive oxygen species, and up-regulation of cell survival ability, which was dysregulated during ketamine induction. In addition, RT-PCR analysis of DOPA-related genes suggests that the compound exerted significant inhibition in the expression of these genes, which were overexpressed during ketamine induction. The current findings provide new insight into the neuroprotective mediation of bioactive factors as a prospective therapy for neurological disorders.

## 1. Introduction

Numerous neuroprotective screening studies have been conducted using traditional medicinal plants in an attempt to discover new therapeutic agents that are devoid of the toxic side effects generally known to be associated with current therapeutic agents. Currently, research is focused more on developing neuroprotective drugs and their derivatives isolated from natural sources, especially phytochemicals with improved neuromodulator activity and minimal systemic toxicity. Among the active phytoconstituents of plant origin, flavonoids are one the best-studied groups of compounds for their neuroprotective effects, and a selected few are presented here as classical examples of their representative structural class. In their study, Li et al. (2012) demonstrated the neuroprotective property of the compound baicalein, isolated from *Scutellaria baicalensis* (Labiatae), against 6-OHD-A-induced Parkinsonism and inhibition of ROS accumulation in rotenone-induced apoptosis in vivo and in vitro [1].

Pinocembrin, a flavonoid isolated from *Pinus heartwood*, *Eucalyptus populus*, *Euphorbia*, and *Sparattosperma leucanthum*, is reported for its neuroprotective effect against cerebral ischemic injury and antiexcitotoxic effects [2], enhanced cognition by protecting cerebral mitochondrial structure and function against chronic cerebral hypoperfusion in rats [3]. The flavonol glycoside rutin, its aglycone quercetin, and a related glycoside isoquercetin (quercetin 3-O-glucoside) were shown to possess neuroprotective effects in HT22 hippocampal cells [4]. Quercetin also dose-dependently reduced paralysis in *Caenorhabditis elegans* by decreasing the amount of aggregated proteins [5] and antagonized the high glucose-induced damage of Schwann cells by inducing autophagy [6]. Puerarin is a subclass of flavonoid compounds obtained from *Pueraria lobata* and was noted for upregulation of phosphorylation of Akt in MPP^+^-induced cytotoxicity in SH-SY5Y cells [7]; inhibition of MPP^+^-induced nuclear translocation of p53; expression of Bax; and caspase-3-dependent programmed cell death [8], neuronal protection [9], and inhibition of autophagy via the Akt signaling pathway and oxidative stress in STZ-induced SAD mice [10].

Pandy et al. (2012) demonstrated the antidopaminergic effect of *Morinda citrifolia* in mice [11]. Several research groups, such as Zhanget et al. (2004), Bigoniya and Rana (2005), and Kothari et al. (2010), identified and reported several medicinal plants exhibiting antipsychotic and antidepressant activities using in vivo models [12,13,14]. In view of the relative promising activity of the flavonoid group of molecules from medicinal plants as potent neuroprotective agents, in our previous study, we isolated and characterized a bioactive compound, 3-(3,4-dimethoxy phenyl)-1-(4-methoxy phenyl)prop-2-en-1-one (structural analog of flavonoid), from the medicinal plant *Celastrus paniculatus* (CP) and reported its neuroprotective activity in vivo, with particular reference to the cholinergic, dopaminergic, glutamatergic, and ATPase systems [15,16,17,18]. In continuation of the earlier studies, the current study aimed to determine the in vitro neuroprotective activity of this bioactive compound against ketamine-induced neurotoxicity in PC 12 cell lines. Clozapine, an atypical antipsychotic drug that has proved to be a strong neuroprotective agent, was used throughout the study as a reference drug [19,20].

## 2. Materials and Methods

### 2.1. Procurement of Chemicals

All chemicals used in the present study were analytical grade and obtained from the following scientific companies: Sigma (St. Louis, MO, USA), Fisher (Pittsburg, PA, USA), Merck (Mumbai, India), Ranbaxy (New Delhi, India), and Qualigens (Mumbai, India). In addition, various in vitro assay kits such as MTT, LDH, DCF-DA, and PCR were purchased from Medox Biotech (India Pvt. Ltd., Chennai, India), Sigma (St. Louis, MO, USA), Fisher (Pittsburg, PA, USA), and Invitrogen (Thermo Fisher Scientific, Waltham, MA, USA).

### 2.2. Source of Plant Constituent and Cell Lines

The bioactive principle used in the current study for the determination of neuroprotective activity was previously isolated, characterized from the seeds of the medicinal plant *Celastrus paniculatus* and was reported for in vivo neuroprotective activity with particular reference to the cholinergic, dopaminergic, glutamatergic, and ATPase systems [15,16,17,18]. PC 12, a cell line derived from pheochromocytomas of the rat adrenal medulla, has an embryonic origin from the neural crest, has a mixture of neuroblastic properties, and has been reported in the study of expressions of catecholamines, dopamine, and norepinephrine. Furthermore, PC 12 cells have been used extensively as a model to study pharmacologic profiles of natural or synthetic compounds against drug-induced catecholamine dysregulation in vitro. The PC 12 cell line was purchased from National Center for Cell Sciences, Pune. The cells were maintained in Dulbecco’s Modified Eagle medium (DMEM) supplemented with 8% horse serum, 2% FBS, 100 µg/mL penicillin, and 100 µg/mL streptomycin in a water-saturated atmosphere of 5% CO_2_ at 37 °C. The medium was changed every three days, and the confluent cells were passaged by trypsinization weekly.

### 2.3. Toxicity Studies

The study was conducted in accordance with OECD guidelines (Testing of Chemical Number 423). Healthy PC 12 cell lines were used in this investigation. Cell lines were treated with different concentrations, i.e., from 20 µg/mL to 600 µg/mL of the title compound and clozapine and observed for 48 h from the time of administration. The median non-toxic concentration (MNTC) and cytotoxic concentration (CC50) were determined. The minimum concentrations of the test and reference compounds that did not show any toxic effects on healthy PC 12 cell lines were selected for further in vitro analyses.

### 2.4. Experimental Design (In Vitro)

Group I: Healthy cell lines

Group II: Exposed to different concentrations of ketamine (10–50 µg/mL)

Group III: Ketamine-treated cell lines treated with different concentrations of clozapine (reference drug) (10–50 µg/mL) for 2 h after treatment with ketamine

Group IV: Ketamine-treated cell lines treated with different concentrations of plant compound (10–50 µg/mL) for 2 h before ketamine treatment

### 2.5. Determination of Tissue Culture Inhibitory Concentration (IC50)

The PC 12 cell lines were seeded in a microtiter plate, treated with different concentrations of 10-fold dilutions of ketamine ranging from 10^1^ to 10^8^, and incubated at 37 °C for 24 h. The inhibitory concentration of cell viability was determined using a cytotoxic assay, and the morphological images of the cell line were observed using an inverted microscope.

### 2.6. Determination of Tissue Culture Effective Concentration (EC50)

The effective concentration of the plant compound and clozapine was determined using PC 12 cell lines. The PC 12 cell lines were seeded in a microtiter plate and treated with different concentrations of 10-fold dilutions of clozapine (ranging from 10^1^ to 10^8^) for 2 h after treatment with ketamine at different concentrations, or with plant compound for 2 h before ketamine treatment at different concentrations, and incubated at 37 °C for 24 h. It is postulated that in order to determine the therapeutic efficacy of bioactive compounds, pretreatment with the new compound against the drug-induced dysregulations is compulsory. If the compound exhibits significant activity, the percentage of activity is compared with that of reference drugs already proven for the activity through in vitro or in vivo models. After getting far better or equal results with the compound when compared with reference drug, the post-treatment method can be determined. Earlier reports demonstrated that pretreatment with different concentrations of the bioactive compounds for 6 h followed by 6-hydroxydopamine (6-OHDA), a neurotoxicant, exhibited a significant antiapoptotic property in PC 12 cells [21]. It was also mentioned that clozapine in posttreatment counteracts the ketamine-induced decrease in NSC viability in correlation with decreased apoptosis and autophagy [22]. In light of this connection, pretreatment with bioactive compounds before ketamine treatment and post-treatment with clozapine after ketamine treatment has followed during the conduction of in vitro studies. The effective concentration on cell viability was determined using a cytotoxic assay, and the morphological images of the cell line were observed using an inverted microscope.

### 2.7. MTT Assay

The cell viability of PC 12 cell lines during pretreatment with the compound was assessed using a Medox Bio MTT assay kit according to the method of Kumar et al. (2018). A fixed number (5 × 10^6^) of exponentially growing cells were seeded into 96-well microtiter plates and allowed to grow. Twenty-four hours later, several of the individual cell cultures were used for the experiments as explained in the experimental design, followed by incubation at 37 °C for 24 h. The cell viability was measured using the MTT assay. The treated cells were incubated with 10 µL of MTT for 3 h followed by 10 µL solubilization solution and mixed thoroughly using a pipette. The MTT medium was carefully aspirated from the wells, and formazan dye was eluted using DMSO. Absorbance was measured using a spectrophotometer (microplate reader) at a wavelength of 570 nm [23]. The percentage of cell viability was calculated by subtracting the percentage of cell viability over control cells.

### 2.8. LDH Assay

LDH released in the medium was measured according to the procedure of the Pierce LDH Cytotoxicity assay kit (88953) method, as described by Soyingbe et al. (2018). A fixed number (5 × 10^5^) of exponentially growing cells were seeded into several individual 25-cm^2^ culture T-flasks and allowed to grow. Twenty-four hours later, several of the individual cell cultures were used for the experiments as described in the experimental design, followed by incubation at 37 °C for 24 h. At the end of the treatment, the whole medium from the cell culture flask of the control and treated groups was removed and collected separately. The tubes containing media were centrifuged at 1500 rpm for 10 min, and 50 μL of the medium was transferred to individual tubes containing PBS buffer, followed by 10 min incubation at 37 °C, the addition of 50 µL of reaction mixture, incubation at room temperature for 30 min, and the addition of 50 µL of stop solution to each well, mixed by gentle tapping. The absorbance was measured at 490 nm [24].

### 2.9. DCFH-DA Assay

The intracellular ROS levels were estimated using an ab113851 DCFDA ROS detection assay kit according to the method of Heo et al. (2018). The exponentially growing cells were seeded into 96-well microtiter plates at 2.5 × 10^4^ cells/well and were allowed to attach for 24 h at 37 °C, and 100 µL of 1× PBS buffer was added. The cells were then washed with 1× PBS buffer. The 1× buffer was removed, and the cells were stained by adding 50 µL of the diluted DCFDA solution. Then the cells were incubated for 45 min at 37 °C in the dark. The fluorescence intensity was measured using a fluorescence microtiter ELISA plate reader at excitation and emission wavelengths of 485 nm and 535 nm, respectively [25].

### 2.10. Clonogenic Assay

The clonogenic cell survival was measured using colony forming assay on the PC 12 cell lines according to the method of McDonald et al. (2018) [26]. A fixed number (5 × 10^5^) of exponentially growing cells were seeded into several individual 25-cm^2^ culture T-flasks and were allowed to grow. Several individual cell cultures were randomly divided 48–72 h after culture initiation, used for experiments as described above, and allowed to incubate at 37 °C for 24 h. At the end of various experiments, cells from the above groups were trypsinized, cells in the single cell suspension were counted using an Olympus INT-2 inverted microscope, and an appropriate number of viable cells were seeded into culture Petri dishes in triplicate and were left undisturbed for 3–4 days for colony formation. The colonies formed were then washed with 1× PBS buffer. The 1× PBS buffer was removed, and the cells were fixed with formaldehyde (4%) prepared in methanol and were stained with crystal violet (0.5%) for 30 min. The viable colonies were counted by using an Olympus INT-2 inverted microscope. The plating efficacy and survival fraction were then calculated as follows.

Plating efficacy (PE) = (Number of colonies counted/Number of cells seeded) × 100.

Survival fraction (SF) = (PE of the treated sample/PE of control) × 100.

### 2.11. Isolation of RNA

RNA extraction was carried out using a single-step method described by Villa Rodriguez (2018) with TRIzol Reagent (Invitrogen) [27]. The cell line pellet was added with 750 µL of TRIzol reagent and incubated for 15 min at 37 °C. Then 250 µL of chloroform was added to it for phase separation. The contents were mixed by inversion for 15 s and allowed to stand at room temperature for 15–30 min. By this time, the solution was separated into two phases, and this was centrifuged at 12,000 rpm for 15 min at 4 °C. The aqueous phase containing RNA was transferred to an RNase-free microcentrifuge tube, mixed with 500 µL of isopropanol, incubated for 15–30 min at room temperature, and centrifuged at 12,000 rpm for 10 min at 4 °C. The supernatant was discarded, and the RNA pellet was washed with 80% chilled ethanol twice at 10,000 rpm for 10 min at 4 °C. After discarding the supernatant, the RNA pellet was air-dried and dissolved in 20 µL of RNase-free water. 

### 2.12. Complementary DNA Synthesis (cDNA)

Complementary cDNA synthesis was carried out using reverse transcriptase enzyme for the RNA-isolated groups as per the method described by Suzuki et al. (2018) [28]. An amount of 2 µL of FQW and 1 µL of RHP was added to 5 µL of RNA, and the RNA was denatured by heating at 95 °C for 5 min before snap cooling on ice, along with the 4 µL of 5× RT buffer, 4 µL of ribolax, and 2 µL of 10 mM dNTPs mix. The reaction was allowed to anneal by keeping it at room temperature for 5 min, and cDNA synthesis was carried out by adding 2 µL of RT at 42 °C for 90 min. The RT was stopped by heating the mixture at 75 °C for 10 min, and the cDNA thus prepared was stored at −20 °C.

### 2.13. Polymerase Chain Reaction of cDNA Synthesized Groups

The synthesized cDNA was subjected to polymerase chain reaction in order to amplify the dopaminergic (DOPA) genes using a specific set of primers such as TH (FP: GAGTTTGACCCTGACCTGGAC; RP: CTCACCCTGCTTGTATTGGAA), DDC (FP: CGCAAGTGAATTCCGAAGGA; RP: ACCTGGCGTCCCTGAAT), DBH (FP: ACCGGCTACTGCACAGACAAG; RP: TCCTGCCCGTCAGGTGTGT), and VMAT2 (FP: CGAGCATCTCTTATCTCATTGGA; RP: ATAGCCACCTTCCCATTTTGTG). β-actin (ACTB) (FP: GGGCATCCTGACCCTCAAG; RP: TCCATGTCGTCCCAGTTGGT) was used as an internal control. The reaction was carried out in a final volume of 50 µL in 200 µL PCR tubes by adding the following reagents: 10× Taq DNA polymerase buffer (5 µL), 10 nM dNTPs mix (2 µL), MgCl_2_ (3µL), FP (1 µL), RP (1 µL), cDNA (2 µL), and Taq DNA polymerase (1 µL). The total volume of the reaction mixture was increased to 50 µL with nuclease-free water. The tubes were then spun for 20 s, and PCR was carried out in an Eppendorf Master Cycler. A heated lid was used to prevent condensation on the walls of the tube. The reaction conditions were standardized for the amplification of genes. The amplified products were analyzed using 1% agarose prepared in 1× TBE containing ethidium bromide at a final concentration of 0.5 µg/mL. The PCR products were mixed with 1 µg of 6× gel loading dye and electrophoresed at 60 volts for 90 min. After the electrophoresis, the product profile was visualized in a UV transilluminator, and images were taken using a UVP Gel Doc IT system [29].

### 2.14. Densitometric Analysis

The intensity of DOPA-related genes expressed in the obtained gel image was subjected to densitometric analysis using image quant software (GE Healthcare) for normalization of DOPA gene expression in the PCR reaction of PC 12 cells, according to the protocol given by Rasband (2018) [30].

### 2.15. Statistical Analysis

Values of the measured parameters were expressed as means ± SEM. One-way- ANOVA (F value) was used to test the significance of the difference among more than two arithmetic means, followed by Dunnett’s test to test the difference between each set of two means. Statistical significance was considered at *p* values < 0.05. All statistical analyses were processed using Statistical Program of Social Sciences (SPSS) for Windows, version 20.0.

## 3. Results

### 3.1. Determination of Tissue Culture Inhibitory Concentration of Ketamine, Effective Concentration of Clozapine and Compound

**Figure 1:**
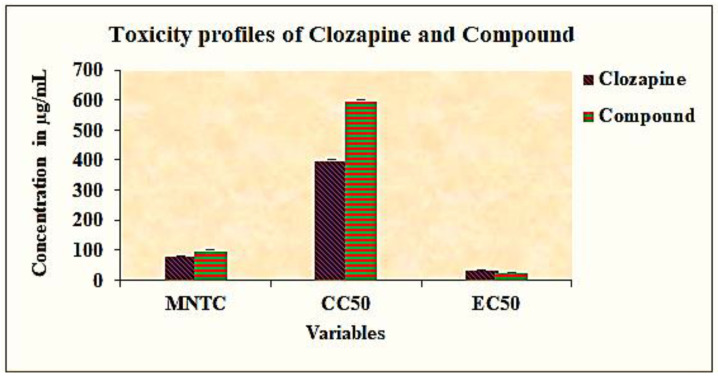
Graphical representation of MNTC, CC50, and EC50 of compound and clozapine.

Prior to the evaluation of the neuroprotective property of the bioactive compound, the median nontoxic concentration (MNTC) and cytotoxic concentration (CC50) of the plant compound and clozapine were determined by incubating PC 12 cell lines with different concentrations of plant compound and clozapine ranging from 20 to 600 µg/mL for 48 h. The MNTC was determined as 100 µg/mL and 80 µg/mL for the compound and clozapine; the CC50 was determined as 600 µg/mL and 400 µg/mL for the compound and clozapine respectively. The inhibitory concentration (IC50) of ketamine was determined by incubating PC 12 cell lines with different concentrations of ketamine ranging from 10 to 50 µg/mL. The IC50 of ketamine was determined as 10.40 µg/mL (Table 1). By contrast, the effective concentration of the plant compound and the reference compound clozapine was determined by incubating the cell lines with plant compound before treatment with ketamine and with clozapine and after treatment with ketamine. The EC50 values of the plant compound and clozapine were determined as 14.67 µg/mL and 24.67 µg/mL, respectively. The MNTC, CC50, and EC50 of the plant compound and clozapine are shown in Figure 1.

### 3.2. Cytotoxicity of the Compound by MTT Assay

**Figure 2:**
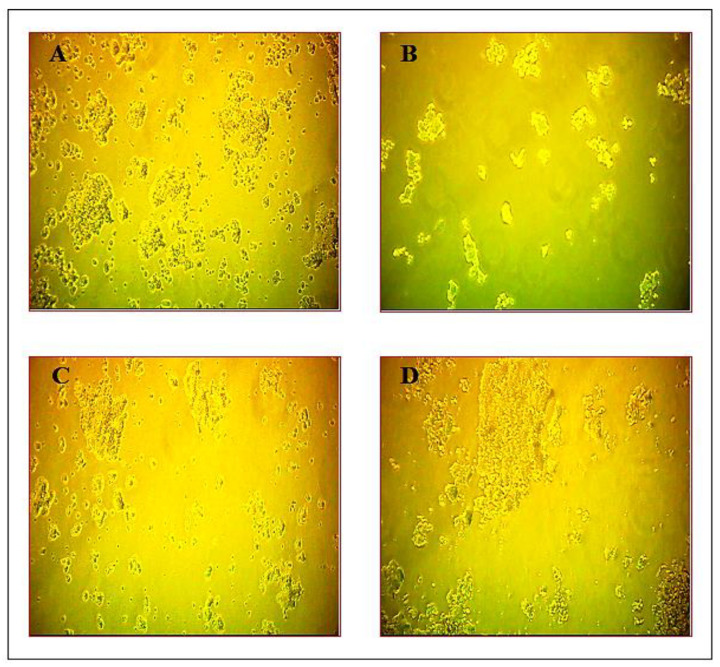
Effect of plant compound and clozapine on cell viability of PC 12 cell lines during in vitro ketamine treatment. (**A**) PC 12 cell line (control). (**B**) PC 12 cell line treated with ketamine for 12 h. (**C**) PC 12 cell line treated with clozapine for 2 h after ketamine treatment. (**D**) PC 12 cell line treated with plant compound for 2 h before ketamine treatment. Scale: 100 μm.

**Figure 3:**
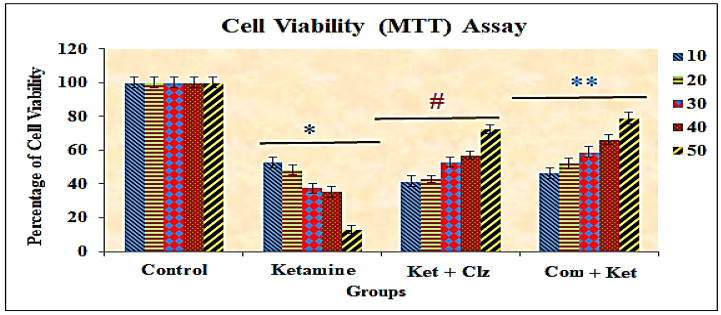
Alterations in cell viability in PC 12 cell lines exposed to ketamine and protective effects of plant compound and clozapine. All the values are expressed as means ± SD of six individuals. * Significant at *p* < 0.05 compared with control; # and ** significant at *p* < 0.05 compared with ketamine-treated group; 10–50: concentration in µg/mL of ketamine, clozapine, and compound.

The neuroprotective effect of the plant compound against ketamine-induced cellular damage was determined by calculating the percentage of MTT reduction in PC 12 cells. When PC 12 cells were exposed to different concentrations of ketamine of 10–50 µg/mL for 24 h, cell viability was reduced significantly from 50.4 ± 3.2 to 14.1 ± 2.2 compared with the control group viability. Approximately 90% of cell death was observed in PC 12 cell lines treated with 50 µg/mL concentration of ketamine. In order to study the neuroprotective effects of plant compound, PC 12 cells were treated with different concentrations (10–50 µg/mL) of the compound for 2 h before exposure to ketamine for 24 h. Pretreatment with plant compound protected PC 12 cells against ketamine-induced damage in a dose-dependent manner. Similarly, post-treatment with clozapine significantly protected the PC 12 cells against ketamine-induced cytotoxicity. Approximately 80% of cell viability was observed with pretreatment with the plant compound when compared with the normal control group, and 72% of cell viability was observed with clozapine. The protective effect of the plant compound was even stronger than that of clozapine (reference drug). Treatment with the plant compound significantly ameliorated the morphological damage and cell viability of PC 12 cell lines compared with the ketamine-treated PC 12 cell line. No significant cell morphological changes were observed in the compound-treated cell line group, and the compound did not exhibit cytotoxicity (Figure 2 and Figure 3).

### 3.3. Lactate Dehydrogenase (LDH) Assay

**Figure 4:**
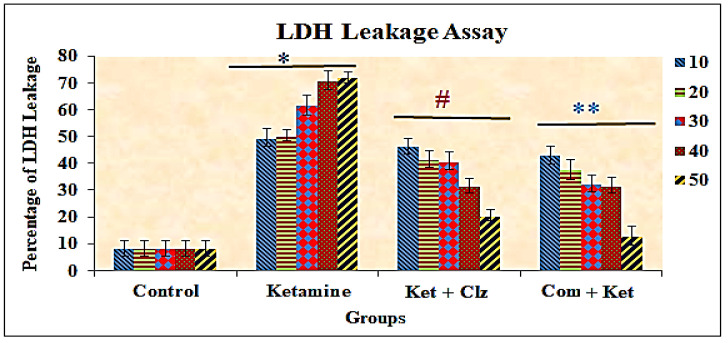
Protective effects of plant compound and clozapine on LDH activity in the culture medium of PC 12 cell lines treated with plant compound and clozapine. All the values are expressed as means ± SD of six individuals. * Significant at *p* < 0.05 compared with control; # and ** significant at *p* < 0.05 compared with ketamine treated group; 10–50: concentration in µg/mL of ketamine, clozapine, and compound.

The LDH activity in the medium increased proportionately when the cells were treated with different concentrations of ketamine when compared with the control group. The LDH released into the medium was determined as approximately 50% at a 10 µg/mL ketamine concentration. The activity levels of LDH were significantly reduced with pretreatment with the plant compound, determined as 12% at a 50 µg/mL concentration of plant compound. By contrast, the LDH activity levels were decreased by 23.58% in the medium with treatment with 50 µg/mL clozapine when compared with ketamine treatment. The increased activity levels of LDH in the medium were reversed with the pretreatment of plant compound and clozapine compared with the ketamine-treated group (Figure 4).

### 3.4. Measurement of Intracellular ROS

**Figure 5:**
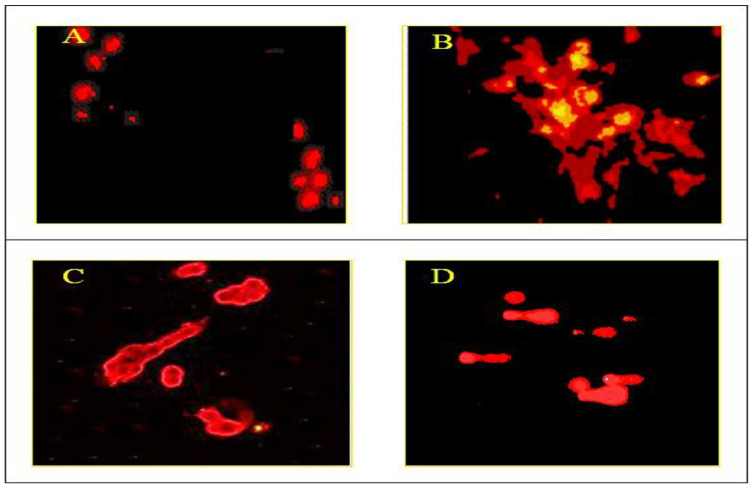
Effect of plant compound and clozapine on the production of ROS in PC 12 cell lines during ketamine induction in vitro. (**A**) PC 12 cell line (control). (**B**) PC 12 cell line treated with ketamine for 12 h. (**C**) PC 12 cell line treated with clozapine for 2 h after ketamine treatment. **(D**) PC 12 cell line treated with compound for 2 h before ketamine treatment. Scale: 100 μm.

**Figure 6:**
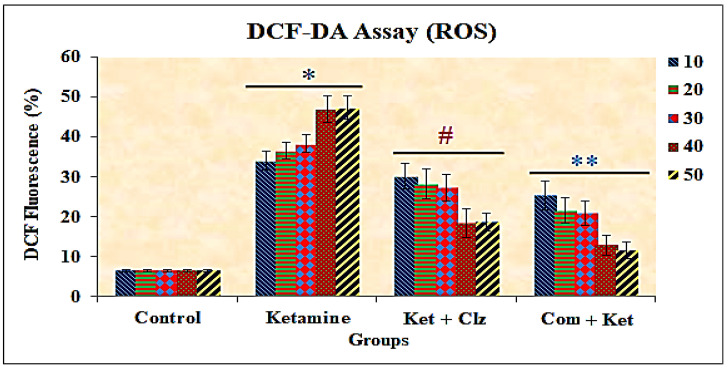
Protective effects of plant compound and clozapine on ROS levels in PC 12 cell lines exposed to ketamine in vitro. All the values are expressed as means ± SD of six individuals. * Significant at *p* < 0.05 compared with control; # and ** significant at *p* < 0.05 compared with ketamine treated group; 10–50: concentration in µg/mL of ketamine, clozapine, and compound.

The results of the DCF-DA assay indicated a significant increase in the levels of ROS in the ketamine-treated cells when compared with the control, and decreased levels were observed in the cells with pretreatment with the plant compound and clozapine compared with the ketamine treated group (Figure 5 and Figure 6). A dose-dependent decrease in ROS levels was observed during pretreatment with the plant compound, and the percentage of ROS decrease was significantly greater than that of the reference compound clozapine.

### 3.5. Clonogenic Assay

**Figure 7:**
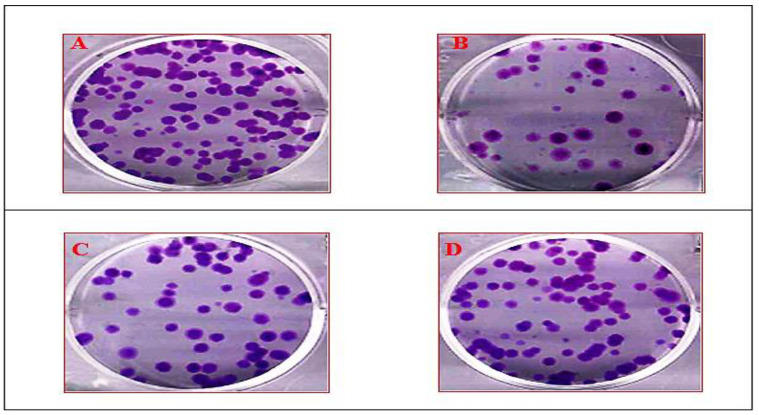
Clonogenic assay of PC 12 cell lines during treatment with ketamine, clozapine, and plant compound. (**A**) PC 12 cell lines after seeding 100 cells/plate. (**B**) PC 12 cell lines treated with ketamine after seeding 100 cells/plate. (**C**) PC 12 cell lines treated with clozapine for 2 h after treatment with ketamine after seeding 100 cells/plate. (**D**) PC 12 cell lines treated with compound for 2 h before ketamine treatment after seeding 100 cells/plate. Scale: 100 μm.

**Figure 8:**
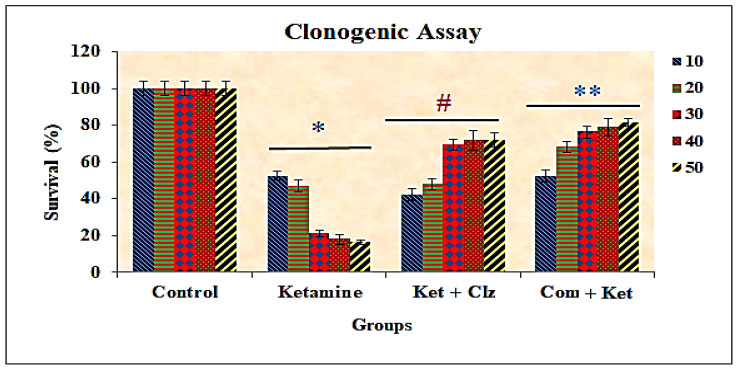
Effects of plant compound and clozapine on clonogenic assay (cell survival ability analysis) of PC 12 cell lines during ketamine induction in vitro. All the values are expressed as means ± SD of six individuals. * Significant at *p* < 0.05 compared with control; # and ** significant at *p* < 0.05 compared with the ketamine treated group; 10–50: concentration in µg/mL of ketamine, clozapine, and compound.

Colony formation assay provides an appropriate measure of the long-term effects of potential therapeutic agents, assessing the ability of cells to retain proliferative potential after treatment. The clonogenic assay in the present study indicated a reduction of colony formation capacity in PC 12 cell lines treated with various concentrations of ketamine compared with the control group. By contrast, a significant enhancement of cell proliferation was observed in PC 12 cells treated with plant compound and clozapine compared with the ketamine treated group (Figure 7 and Figure 8).

### 3.6. Semi-Quantitative RT-PCR Analysis

**Figure 9:**
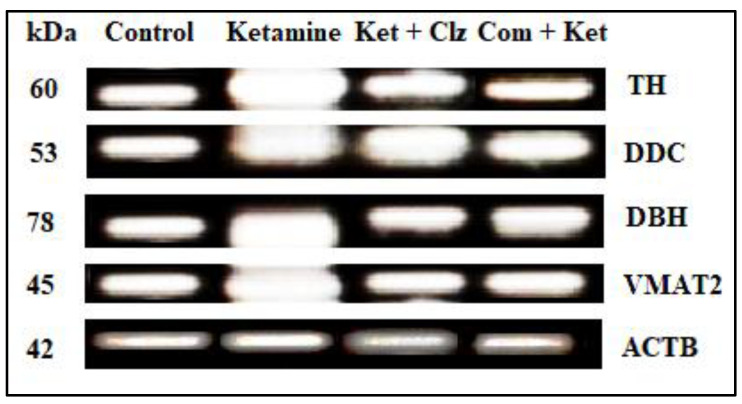
Effects of plant compound and clozapine on the expression of DOPA genes during ketamine treatment in vitro. TH: Tyrosine hydroxylase. DDC: Dopamine decarboxylase. DBH: Dopamine beta hydroxylase. VMAT2: Vesicular monoamine transporter 2. ACTB: β-actin (internal control).

RNA extraction was carried out using a single step with TRIzol reagent (Invitrogen). The total RNA extracted from cell culture was subjected to cDNA synthesis by reverse transcriptase enzyme (M-MuLV) using a specific set of primers for amplification of dopaminergic genes. Denaturation at 95 °C for 5 min and cDNA synthesis at 42 °C for 90 min showed good amplification on PCR. In addition, 35 cycles of PCR with an annealing temperature of 49 °C at a 1.5 mM concentration of MgCl_2_ was found to be optimum for the amplification of DOPA genes. The size of the amplified products of DOPA genes such as TH (222 bp), DDC (81bp), DBH (606bp), and VMAT2 (326bp) was analyzed by agarose gel electrophoresis by employing a standard DNA molecular size marker (Fermentas 1 Kb plus ladder). Normalized amplification was observed in the cell control; overexpression was observed in the DOPA-related genes and the elevated level of expression was observed in these four genes in the ketamine-treated group. The observed overexpression of DOPA genes was reduced during treatment with the plant compound and clozapine, indicating that the compound inhibits the overexpression of DOPA genes on par with the clozapine reference compound. No significant differences in β-actin expression were found between untreated and treated groups (Figure 9).

### 3.7. Densitometric Analysis

**Figure 10:**
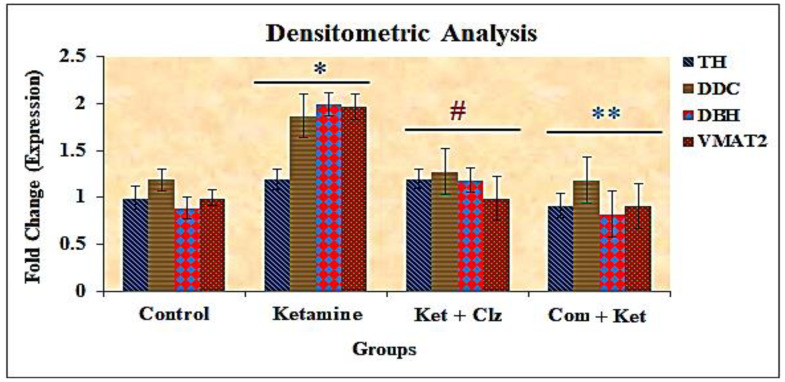
Effects of plant compound and clozapine on the expression of DOPA genes during ketamine-induced alterations in vitro (fold change). All the values are expressed as means ± SD of six individuals. * Significant at *p* < 0.05 compared with control; # and ** significant at *p* < 0.05 compared with ketamine-treated group. TH: Tyrosine hydroxylase. DDC: Dopamine decarboxylase. DBH: Dopamine beta hydroxylase. VMAT2: Vesicular monoamine transporter 2.

To further investigate the protective effect of the compound on the dopamine system, the intensity of DOPA-related genes expressed in the obtained gel image was subjected to densitometric analysis using image quant software (GE Healthcare) for normalization of DOPA genes expression in the PCR reaction of PC 12 cells. The results indicated that genes such as TH (60 kDa), DDC (53 kDa), DBH (78 kDa), and VMAT2 (45 kDa) were formed into dimers and expressed significantly in the ketamine group, as evidenced by densitometric values for TH (60 kDa; 1.19), DDC (53 kDa; 1.86), DBH (78 kDa; 1.99), and VMAT2 (45 kDa; 1.96) when compared with the normal control group. However, the overexpression of the above enzymes reverted to the control values with pretreatment with the compound when compared with the ketamine-treated group, as evidenced by the densitometric values, viz., 0.99, 1.19, 0.88, and 0.99 for TH (60 kDa), DCC (53 kDa), DBH (78 kDa), and VMAT2 (45 kDa), respectively (Figure 10).

## 4. Discussion

Recently, there has been intense interest in the potential use of flavonoids to modulate neuronal function and protection against disorders related to neurodegeneration. The use of flavonoid-rich plant or food extracts in humans and animal dietary supplementation studies showed improvements in cognitive function, possibly by protecting vulnerable neurons, enhancing existing neuronal function, or stimulating neuronal regeneration [31]. Their neuroprotective potential has been shown to ameliorate both oxidative [32] and Ab-induced neuronal death models [33]. Evidence also exists for the beneficial and neuromodulatory effects of flavonoid-rich *Ginkgo biloba* extracts, particularly in connection with age-related dementia and Alzheimer’s disease [34]. Furthermore, individual flavonoids, such as the citrus flavanone tangeretin, have been observed to maintain nigro-striatal integrity and functionality following lesioning with 6-hydroxydopamine, suggesting that they may serve as a potential neuroprotective agent against the underlying pathology associated with Parkinson’s disease [35]. In addition, flavonoids may exert beneficial effects on memory, prevent cognitive losses associated with aging, and even reverse certain age-related declines [36].

The present study is the first report to evaluate in vitro neuroprotective effects of a bioactive compound against cell death induced by ketamine toxicity in the PC 12 cell line. Treatment of PC 12 cell lines with ketamine caused a significant decrease in cell viability in a dose-dependent manner. Previous studies have shown that ketamine has pro-apoptotic properties that induce neuroapoptosis in the developing brain and cultured neurons in vitro [37] as well as inhibition of the proliferation of neurons in vitro [38]. Furthermore, ketamine led to significant ultrastructural abnormalities (including mitochondrial fragmentation, a decrease in Golgi and rough endoplasmic reticulum, and an increase in autophagosomes). Ketamine might also alter other cell physiological activities, such as neuronal receptor expression and the structure and branching of neurons, dendrites, and synaptogenesis, eventually resulting in impaired neuronal function. Pretreatment with the plant compound decreased ketamine-induced cytotoxicity and offered significant protection, as evidenced by enhanced cell viability and decreased neuronal damage and cell necrosis. The present findings also suggest that the percentage of recovery of cell viability is greater with pretreatment with the plant compound than with clozapine (Figure 2 and Figure 3).

The estimation of lactate dehydrogenase in cell culture medium is known to provide a quantitative basis for the loss of cell viability and is frequently used to estimate the cytotoxic effects of various drugs [39]. Since LDH is released only when the cell membrane is damaged, it has been used in the past as an indirect indicator of the loss of cell membrane integrity [40]. An LDH leakage assay was used in the present investigation to confirm the results of the MTT assay as well as to study whether cell membrane damage could possibly be the mode of cell death induced by ketamine in PC 12 cells. Treatment of PC 12 cells with ketamine resulted in a significant, concentration-dependent increase in the LDH activity levels in the culture medium, which supported the findings of the MTT assay. Earlier studies provided conclusive evidence that ketamine causes a loss of membrane integrity, leading to LDH leakage into the culture medium [41]. It was reported that ketamine activated the mitochondrial apoptotic pathway and induced cell apoptosis in cultured cortical neurons through the NMDA receptor NR1 subunit [42]. Increased NMDA receptors were reported to alter the ion and water homeostasis in the cell and thus cause cell membrane damage [43], which is the hallmark of necrosis. Cell necrosis following ketamine treatment was confirmed by a marked increase of lactate dehydrogenase (LDH) leakage in neuronal cells as well as human hepatoma HepG2 cells [44,45]. Thus, cell necrosis might also contribute to ketamine’s toxic effects. PC 12 cells are dopaminergic cells, and a loss of integrity in the plasma membrane would lead to dopamine leakage without activation of Ca^2+^ dependent dopamine release. Furthermore, ketamine has also been reported to promote spontaneous dopamine release, a process independent of extracellular Ca^2+^ [46]. The LDH leakage was significantly reduced in a dose-dependent manner in PC 12 cell lines with pretreatment with the compound compared with the ketamine treated group, which suggests that the compound protects the architectural damage of cell membrane caused by ketamine cytotoxicity. It was also observed that LDH leakage was reduced more during treatment with the compound than with the reference drug clozapine (Figure 4).

Reactive oxygen species have also been proposed to play a vital role in the pathology of many neurodegenerative diseases [36]. Accumulating evidence indicates that flavonoids are effective in blocking oxidant-induced neuronal injury [47] and are believed to act by modulating a number of protein kinase and lipid kinase signaling cascades, such as the PI3 kinase (PI3 K)/Akt, tyrosine kinase, protein kinase C (PKC), and mitogen-activated protein kinase (MAP kinase) signaling pathways [47]. Inhibitory or stimulatory actions of these pathways are likely to profoundly affect neuronal function by altering the phosphorylation state of target molecules, leading to changes in caspase activity and/or gene expression [48]. Flavonoids have been observed to block oxidative induced neuronal damage by preventing the activation of caspase-3, providing evidence in support of their potent antiapoptotic action [49]. A concentration-dependent elevation in intracellular ROS levels after treatment with ketamine in the present study indicates that oxidative stress could be an important mechanism by which ketamine exerts its toxic activity in PC 12 cells. On the other hand, a significant reduction was observed in the formation of ROS levels in PC 12 cell lines during pretreatment with the compound, and the ROS levels were completely drawn back to the control levels. The depletion in ROS levels was also observed during the treatment with clozapine, and the depletion was greater with treatment with the plant compound than with clozapine (Figure 5 and Figure 6). Importantly, the bioactive compound not only attenuated ketamine-induced ROS production but also prevented cell death, suggesting that ketamine-induced neuroapoptosis is directly associated with enhanced ROS production.

The clonogenic survival assay is an in vitro cell survival assay that is used to test the cytotoxic potential of a test agent. Treatment of PC 12 cells with ketamine caused a dose-dependent reduction in cell survival in comparison with the vehicle-treated controls. By contrast, the clonogenic survival was significantly elevated in the PC 12 cell line with pretreatment with the compound when compared with ketamine treatment. These results indicate that the compound protects the PC 12 cell line against ketamine-induced alterations in the clonogenic survival property in vitro, and the survival percentage was greater than the reference drug clozapine (Figure 7 and Figure 8).

From the semi-quantitative RT-PCR analysis, it was observed that overexpression/ dimerization occurred in the DOPA-related genes, such as TH, DDC, DBH, and VMAT2, during ketamine treatment, and the expression was significantly elevated when compared with the control group. It is noteworthy that increased DA contents depended on the upregulation of mRNA levels in DA-related genes. TH is the rate-limiting enzyme in DA biosynthesis, and its activity plays an important role in determining dopamine concentrations [50]. We observed that there was significant upregulation of TH, DDC, DBH, and VMAT2 expression following ketamine treatment. Upregulation of TH expression was also found in animals acutely or chronically treated with other abusive drugs, such as morphine and phencyclidine [51]. Our data suggest the possibility that the upregulation of TH, DDC, DBH, and VMAT2 expression represents a common molecular adaptation in the dopamine system. The formation of dimers during the expression of DOPA genes was significantly reduced in the clozapine-treated group. The reduction in the overexpression in these genes was drawn back to the normal condition and no dimer formation was observed with treatment with the compound and clozapine (Figure 9).

Image J densitometry software (version 1.6, NIH, Bethesda, MD, USA) was used for gel band quantitative densitometric analysis. Selected bands were quantified based on their relative intensities such as area and percentage. Fold increase or decrease between control and treated groups were calculated by dividing values of the control (area and percentage) and treated groups. The fold change was calculated by determining the cumulative densities of DOPA genes between the control and treated groups. The observed overexpression in the DOPA-related genes such as TH, DDC, DBH, and VMAT2 during ketamine treatment may be due to the blockade of NMDA receptors and their hypofunction, which has indirectly shown its impact on genes involved in the synthesis of dopamine and its step gradient consecutive enzymes [52]. With pretreatment with the plant compound, the overexpression of DOPA genes observed in the ketamine group was reduced; the reduction occurred in one-fold manner, and the obtained fold change values were near the control values. This indicates that the ketamine-induced NMDA receptor abnormality was downregulated by the compound, and the synthesis of dopamine was normalized by inhibiting the cytotoxic effects of ketamine on the central dopaminergic system (Figure 10).

## 5. Conclusions

In conclusion, the results from the in vitro assays in this study demonstrate the neuroprotective effects of the bioactive compound 3-(3,4-dimethoxy phenyl)-1-(4-methoxy phenyl)prop-2-en-1-one and add insight into its mechanism of action in ketamine-induced cytotoxicity and neurotoxicity. In addition to the antioxidant actions of the plant compound, the present findings suggest that the plant compound impedes dopaminergic hyperactivity by downregulating the overexpression of dopamine-related genes that occurs during ketamine-induced cytotoxicity. Hence, the information gained from the present study can be used for proposing a better pharmacological tool to treat neurotoxicity and related disorders.

## 6. Significance Statements

From the findings, it was observed that ketamine led to significant ultrastructural abnormalities, altered the cell physiological activities, activated the mitochondria apoptotic pathway and induced cell apoptosis, regulated oxidative stress-related gene expression and upregulation of antioxidant enzymes, and elevated DA release and DA gene expression in PC 12 cell lines. Treatment with the bioactive compound 3-(3,4-dimethoxy phenyl)-1-(4-methoxy phenyl)prop-2-en-1-one exhibited antagonistic activity against ketamine-induced cellular abnormalities in PC 12 cells. The compound reversed the ultrastructural, physiological, defense, apoptosis, and dopaminergic dysregulations that occurred in PC 12 cell lines during ketamine induction and appears to be a promising therapeutic drug candidate for the treatment of neurotoxicity, having multifactorial etiology.

## Figures and Tables

**Table 1:** Cytotoxic effect of ketamine against PC 12 cell line and determination of IC50.

Concentration (µg/mL)	% of Cell Viability with SD
10	50.40 ± 2.23 *#
20	48.63 ± 1.20 *
30	39.25 ± 1.61 *
40	31.05 ± 1.88 *
50	14.10 ± 1.20 *
Control Cells	100 ± 2.24

All values are expressed as the means ± SD of six individuals. * Significant at *p* ˂ 0.01 compared with control. ^#^ Median Inhibitory Concentration (IC50) of ketamine.

## Data Availability

The data presented in this study are available on request from the corresponding author.

## References

[1] Li X.-X., He G.R., Mu X., Xu B., Tian S., Yu X., Du G.-H. (2012). Protective effects of baicalein against rotenone-induced neurotoxicity in PC 12 cells and isolated rat brain mitochondria. Eur. J. Pharmacol..

[2] Shi L.-L., Qiang G.-F., Gao M., Zhang H.-A., Chen B.-N., Yu X.-Y., Du G.-H. (2011). Effect of pinocembrin on brain mitochondrial respiratory function. Acta Pharm. Sin..

[3] Guang H.-M., Du G.-H. (2006). Protections of pinocembrin on brain mitochondria contribute to cognitive improvement in chronic cerebral hypoperfused rats. Eur. J. Pharmacol..

[4] Habtemariam S. (2016). Rutin as a natural therapy for Alzheimer’s disease: Insights into its mechanisms of action. Curr. Med. Chem..

[5] Regitz C., Marie Dußling L., Wenzel U. (2014). Amyloid-beta (A β1-42)-induced paralysis in Caenorhabditis elegans is inhibited by the polyphenol quercetin through activation of protein degradation pathways. Mol. Nutr. Food Res..

[6] Qu L., Liang X., Gu B., Liu W. (2014). Quercetin alleviates high glucose-induced Schwann cell damage by autophagy. Neural Regen. Res..

[7] Zhu G., Wang X., Wu S., Li Q. (2012). Involvement of activation of PI3K/Akt pathway in the protective effects of puerarin against MPP+-induced human neuroblastoma SH-SY5Y cell death. Neurochem. Int..

[8] Zhou Y., Xie N., Li L., Zou Y., Zhang X., Dong M. (2014). Puerarin alleviates cognitive impairment and oxidative stress in APP/PS1 transgenic mice. Int. J. Neuropsychopharmacol..

[9] Li L., Xue Z., Chen L., Chen X., Wang H., Wang X. (2017). Puerarin suppression of Aβ1-42-induced primary cortical neuron death is largely dependent on ERβ. Brain Res..

[10] Tang H., Song X., Ling Y., Wang X., Yang P., Luo T., Chen A. (2017). Puerarin attenuates myocardial hypoxia/reoxygenation injury by inhibiting autophagy via the Akt signaling pathway. Mol. Med. Rep..

[11] Pandy V., Narasingam M., Mohamed Z. (2012). Antipsychotic-like activity of noni (Morinda citrifolia Linn.) in mice. BMC Complementary Altern. Med..

[12] Zhang Z.-J. (2004). Therapeutic effects of herbal extracts and constituents in animal models of psychiatric disorders. Life Sci..

[13] Bigoniya P., Rana A.-C. (2005). Psychopharmacological profile of hydro-alcoholic extract of Euphorbia neriifolia leaves in mice and rats. Indian J. Exp. Biol..

[14] Kothari S., Minda M., Tonpay S.-D. (2010). Anxiolytic and antidepressant activities of methanol extract of Aegle marmelos leaves in mice. Indian J. Physiol. Pharmacol..

[15] Chintha V., Wudayagiri R. (2021). Isolation and neuroprotective prospective of novel bioactive compound “3-(3,4-dimethoxyphenyl)-1-(4-methoxyphenyl) prop-2-en-1-one” against ketamine-induced cognitive deficits in schizophrenia: An experimental study. Nat. Prod. Res..

[16] Venkataramaiah C., Lakshmi Priya B., Rajendra W. (2020). Perturbations in the catecholamine metabolism and protective effect of “3-(3, 4-dimethoxy phenyl)-1-4 (methoxy phenyl) prop-2-en-1-one” during ketamine-induced schizophrenia: An in vivo and in silico studies. J. Biomol. Struct. Dyn..

[17] Venkataramaiah C., Payani S., Priya B.-L., Pradeepkiran J.-A. (2021). Therapeutic potentiality of a new flavonoid against ketamine induced glutamatergic dysregulation in schizophrenia: In vivo and in silico approach. Biomed. Pharmacother..

[18] Venkataramaiah C. (2020). Modulations in the ATPases during ketamine-induced schizophrenia and regulatory effect of “3-(3, 4-dimethoxy phenyl)-1-(4-methoxyphenyl) prop-2-en-1-one”: An in vivo and in silico studies. J. Recept. Signal Transduct..

[19] Bai O., Wei Z., Lu W., Bowen R., Keegan D., Li X.-M. (2002). Protective effects of atypical antipsychotic drugs on PC12 cells after serum withdrawal. J. Neurosci. Res..

[20] Wei Z.-L., Li X. (2001). Neuroprotective effects of some atypical antipsychotics. Fifth Alz Park Dis Abstr.

[21] Lin C.-M., Lin Y.-T., Lee T.-L., Imtiyaz Z., Hou W.-C., Lee M.-H. (2020). In vitro and in vivo evaluation of the neuroprotective activity of Uncaria hirsuta Haviland. J. Food Drug Anal..

[22] Lundberg M., Curbo S., Bohman H., Agartz I., Ögren S.-O., Patrone C., Mansouri S. (2020). Clozapine protects adult neural stem cells from ketamine-induced cell death in correlation with decreased apoptosis and autophagy. Biosci. Rep..

[23] Kumar P., Nagarajan A., Uchil P.-D. (2018). Analysis of cell viability by the MTT assay. Cold Spring Harb. Protoc..

[24] Soyingbe O.-S., Mongalo N.-I., Makhafola T.J. (2018). In vitro antibacterial and cytotoxic activity of leaf extracts of Centella asiatica (L.) Urb, Warburgia salutaris (Bertol. F.) Chiov and Curtisia dentata (Burm. F.) CA Sm-medicinal plants used in South Africa. BMC Complementary Altern. Med..

[25] Heo J.-R., Lee G.-A., Kim G.-S., Hwang K.-A., Choi K.-C. (2018). Phytochemical-induced reactive oxygen species and endoplasmic reticulum stress-mediated apoptosis and differentiation in malignant melanoma cells. Phytomedicine.

[26] McDonald M., Corde S., Lerch M., Rosenfeld A., Jackson M., Tehei M. (2018). First in vitro evidence of modulated electro-hyperthermia treatment performance in combination with megavoltage radiation by clonogenic assay. Sci. Rep..

[27] Villa-Rodríguez E., Ibarra-Gámez C., de los Santos-Villalobos S. (2018). Extraction of high-quality RNA from Bacillus subtilis with a lysozyme pre-treatment followed by the Trizol method. J. Microbiol. Methods.

[28] Suzuki T., Ikeda H., Mase M. (2018). Restricted viral cDNA synthesis in cell lines that fail to support productive infection by bovine leukemia virus. Arch. Virol..

[29] Rebelo A.-R., Bortolaia V., Kjeldgaard J.-S., Pedersen S.-K., Leekitcharoenphon P., Hansen I.-M., Battisti A. (2018). Multiplex PCR for detection of plasmid-mediated colistin resistance determinants, mcr-1, mcr-2, mcr-3, mcr-4 and mcr-5 for surveillance purposes. Eurosurveillance.

[30] Rasband W.-S. (2018). ImageJ..

[31] Youdim K.-A., Spencer J.-P., Schroeter H., Rice-Evans C. (2001). Dietary flavonoids as potential neuroprotectants. Biol. Chem..

[32] Inanami O., Watanabe Y., Syuto B., Nakano M., Tsuji M., Kuwabara M. (1998). Oral administration of (-) catechin protects against ischemia-reperfusion-induced neuronal death in the gerbil. Free Radic. Res..

[33] Luo Y., Smith J.-V., Paramasivam V., Burdick A., Curry K.-J., Buford J.-P., Butko P. (2002). Inhibition of amyloid-β aggregation and caspase-3 activation by the Ginkgo biloba extract EGb761. Proc. Natl. Acad. Sci. USA.

[34] Bastianetto S., Zheng W.-H., Quirion R. (2000). The Ginkgo biloba extract (EGb 761) protects and rescues hippocampal cells against nitric oxide-induced toxicity: Involvement of its flavonoid constituents and protein kinase C. J. Neurochem..

[35] Datla K.-P., Christidou M., Widmer W.-W., Rooprai H.-K., Dexter D.-T. (2001). Tissue distribution and neuroprotective effects of citrus flavonoid tangeretin in a rat model of Parkinson’s disease. Neuroreport.

[36] Jellinger K.-A. (2001). Cell death mechanisms in neurodegeneration. J. Cell. Mol. Med..

[37] Slikker W., Liu F., Rainosek S.-W., Patterson T.-A., Sadovova N., Hanig J.-P., Wang C. (2015). Ketamine-induced toxicity in neurons differentiated from neural stem cells. Mol. Neurobiol..

[38] Wu Y.-Q., Liang T., Huang H., Zhu Y.-Z., Zhao P.-P., Xu C.-M., Zhou C.-H. (2014). Ketamine inhibits proliferation of neural stem cell from neonatal rat hippocampus in vitro. Cell. Physiol. Biochem..

[39] Nehar D., Mauduit C., Boussouar F., Benahmed M. (1998). Interleukin 1α stimulates lactate dehydrogenase A expression and lactate production in cultured porcine Sertoli cells. Biol. Reprod..

[40] Wolterbeek H.-T., Van der Meer A.-J. (2005). Optimization, application, and interpretation of lactate dehydrogenase measurements in micro well determination of cell number and toxicity. Assay Drug Dev. Technol..

[41] Tan S., Lam W.-P., Wai M.-S., Yu W.-H.-A., Yew D.-T. (2012). Chronic ketamine administration modulates midbrain dopamine system in mice. PLoS ONE.

[42] Wang C., Sadovova N., Fu X., Schmued L., Scallet A. (2005). The role of the N-methyl-D-aspartate receptor in ketamine-induced apoptosis in rat forebrain culture. Neuroscience.

[43] Rothman D.L., Behar K., Hetherington H.P., Hollander J.A.D., Bendall M.R., Petroff O.A., Shulman R.G. (1985). 1H-Observe/13C-decouple spectroscopic measurements of lactate and glutamate in the rat brain in vivo. Proc. Natl. Acad. Sci. USA.

[44] Lee S.-T., Wu T.-T., Yu P.-Y., Chen R.-M. (2009). Apoptotic insults to human HepG2 cells induced by S-(+)-ketamine occurs through activation of a Bax-mitochondria-caspase protease pathway. Br. J. Anesthesiol..

[45] Wang C., Sadovova N., Hotchkis C., Fu X., Scallet A.-C. (2006). Blockade of N-methyl-D-aspartate receptors by ketamine produces loss of postnatal day 3 monkey frontal cortical neurons in culture. Toxicol. Sci..

[46] Okamoto T., Okutani R., Tashiro C. (1996). The dual effect of ketamine on dopamine release from rat pheochromocytoma (PC-12) cells. Masui.

[47] Spencer J.P. (2007). The interactions of flavonoids within neuronal signalling pathways. Genes Nutr..

[48] Williams R.-J., Spencer J.-P., Rice-Evans C. (2004). Flavonoids: Antioxidants or signalling molecules?. Free Radic. Biol. Med..

[49] Spencer J.-P., Schroeter H., Crossthwaithe A.-J., Kuhnle G., Williams R.-J., Rice-Evans C. (2001). Contrasting influences of glucuronidation and O-methylation of epicatechin on hydrogen peroxide-induced cell death in neurons and fibroblasts. Free Radic. Biol. Med..

[50] Flatmark T., Stevens R.-C. (1999). Structural insight into the aromatic amino acid hydroxylases and their disease-related mutant forms. Chem. Rev..

[51] Du Bois T.-M., Hsu C.-W., Li Y., Tan Y.-Y., Deng C., Huang X.-F. (2008). Altered dopamine receptor and dopamine transporter binding and tyrosine hydroxylase mRNA expression following perinatal NMDA receptor blockade. Neurochem. Res..

[52] Hons J., Zirko R., Ulrychova M., Cermakova E., Doubek P., Libiger J. (2010). Glycine serum level in schizophrenia: Relation to negative symptoms. Psychiatry Res..

